# A comparison of malaria prevalence, control and management strategies in irrigated and non-irrigated areas in eastern Kenya

**DOI:** 10.1186/s12936-016-1458-4

**Published:** 2016-08-11

**Authors:** John Muthii Muriuki, Philip Kitala, Gerald Muchemi, Ian Njeru, Joan Karanja, Bernard Bett

**Affiliations:** 1 Department of Public Health, Pharmacology and Toxicology, University of Nairobi, P.O. Box 29053-00625, Nairobi, Kenya; 2 International Livestock Research Institute, P.O. Box 30709-00100, Nairobi, Kenya; 3 Department of Disease Surveillance and Response, Kenyatta National Hospital, P. O. Box 20781-00202, Nairobi, Kenya

**Keywords:** Malaria, Prevalence, Control, Management, Irrigation

## Abstract

**Background:**

This study was conducted in Bura irrigation scheme in Tana River County and the pastoral area in Ijara, Garissa County in the eastern Kenya to establish the knowledge, attitude and practices on malaria transmission, control and management, and determine malaria prevalence and the associated risk factors.

**Methods:**

A cross sectional survey design that involved 493 randomly selected people from 334 households was used between November and December 2013. All the randomly selected people were screened for malaria parasites using rapid diagnostic test (RDT)—Carestart™ malaria HRP2 (pf) kit. A questionnaire was administered to determine potential risk factors and perceptions on malaria exposure within a period of 2 months prior to the survey. Two logistic regression models were fitted to the data; one used the RDT results while the other used data from the questionnaire survey.

**Results:**

Using RDT, the prevalence of malaria was 4.68 % (95 % CI: 1.48–7.88 %) and 0.31 % (−0.30 to 0.92 %) in irrigated and non-irrigated areas, respectively. From the questionnaires, 14.62 % (9.27–19.97 %) and 23.91 % (19.23–28.60 %) of the participants perceived to have had malaria in the irrigated and pastoral areas, respectively. The main malaria control measure was the use of bed nets: average of three nets per household in Bura irrigation scheme and one in Ijara. Artemether–lumefantrine was the main drug of choice mainly in the irrigated area while sulfadoxine–pyrimethamine was likely to be used in the non-irrigated area. Households located >5 km from the nearest health facility had higher prevalence of Plasmodium infection than those located ≤5 km.

**Conclusion:**

The residents of Bura irrigation scheme were more likely to be infected compared to those living in the non-irrigated area of Ijara. However, those in the non-irrigated area were more likely to be treated or use over-the-counter medication for perceived malaria illnesses compared to those in the irrigated area. There is a need, therefore, to formulate effective ways of managing malaria especially in irrigated areas and build capacity on differential diagnosis for malaria, especially in the pastoral areas.

## Background

Malaria is a major public health problem worldwide: approximately 214 million cases occur annually and 3.2 billion people are at risk of infection [1]. In 2015, approximately 438,000 deaths were attributed to malaria, especially in sub-Saharan Africa, where about 90 % of all malaria deaths occur [1]. Malaria is one of the leading causes of morbidity and mortality in Kenya accounting for 30–50 % of all outpatient consultations and 20 % of all admissions to health facilities. An estimated 170 million working days are lost to the disease each year.

Malaria incidence has significantly decreased globally: between 2000 and 2015, malaria incidence decreased by 37 % and mortality by 60 % [2]. This progress has been attributed to increased financing, improved planning and partnerships, innovation, development and strengthening of health systems and economic development. Land use change such as irrigation and the associated agricultural practices could alter the gains in reduction of global malaria burden [3]. Irrigation affects microclimatic conditions that influence the abundance and survivorship of mosquitoes by creating standing water masses, which increases humidity [4, 5]. This could aggravate the transmission of the disease by extending the duration of the transmission season [6] or by removing the seasonal occurrence of the disease [3]. The mechanisms underlying this phenomenon remain poorly understood given the complexity of vector ecology, parasite transmission, population immunity and human behaviour.

The Bura irrigation scheme is infested with *Anopheles gambiae* complex which are efficient malaria vectors [7] although other mosquito species such as *Aedes* and *Culicines* are also likely to colonize. Ijara, a neighbouring pastoral area, is a hotspot of Rift Valley fever and other febrile zoonotic diseases associated with livestock husbandry [8, 9]. These diseases may be confused for malaria. This study investigated the prevalence and risk factors of malaria in Bura irrigation scheme in Tana River County and Ijara, a pastoral area in Garissa County in Kenya as well as levels of knowledge, control and management practices used by the local communities.

## Methods

### Study area

**Fig. 1:**
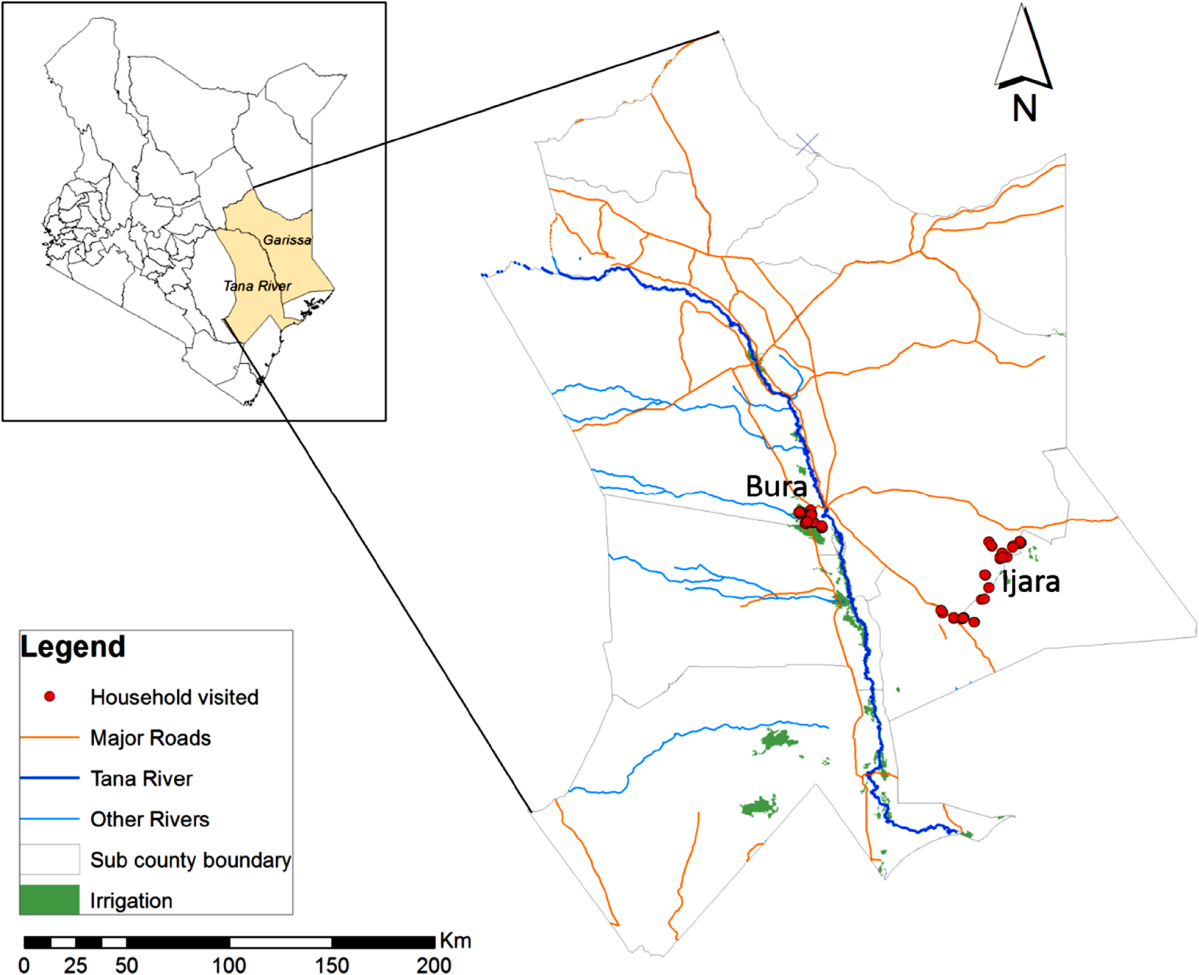
Map of Tana River and Garissa counties showing the study sites: Bura and Ijara

The study was conducted in Bura Irrigation Scheme in Tana River County and Ijara district in Garissa County, Kenya (Fig. 1) between November and December 2013. Both counties are classified as arid and semi-arid areas and they experience short periods of intense malaria transmission during the rainy seasons. The Bura Irrigation Scheme covers about 6250 acres of land out of which, 3000 are under maize cultivation while the rest are mainly used for horticulture (National Irrigation Board, personal interview). According to 2009 Kenya Population and Housing census, the population residing within the irrigation scheme is about 82,545 people. River Tana, the longest river in Kenya, is the source of water for irrigation and it separates Tana River and Garissa Counties. Rainfall is erratic, with rainy seasons in March–May and October–December. Its mean annual rainfall and temperature vary between 400 and 750 mm and 30 and 33 °C, respectively [10]. The area also has a flat terrain which makes it prone to flooding. Malaria is endemic in the area and the major malaria vector species is a mixture of *An. gambiae* sensu stricto and *Anopheles arabiensis* [7].

In Ijara, most people practice nomadic pastoralism. The 2009 Kenya Population and housing census estimated a population of 11,474 people in this area. Temperatures range from 15 to 38 °C with average annual temperatures of 27.2 °C. The area receives bimodal erratic rains ranging between 700 and 1000 mm and averaging 574 mm annually. The rainy seasons are similar to those in Tana River County. Ijara is a hotspot of Rift Valley fever and other febrile zoonotic diseases and the area is usually colonised by various mosquitoes including *Culex* spp. *Aedes* spp., *Anopheles* spp. and *Mansonia* spp. [8, 9, 11].

### Study design

The study used a cross-sectional survey design. Subjects were identified through multistage random sampling technique where households and subjects within households were selected in two successive steps. The sampling frame included all the households in each of the study site. An initial list of the households in Bura was obtained from the National Irrigation Scheme at Bura. This was refined during the initial reconnaissance trips by removing or adding households that had moved out or joined the scheme. A list of all households in Ijara was obtained through door-to-door enumeration. Households were randomly selected from the sampling frame followed by another random selection of up to 5 individuals (who were over 5 years old) within the selected households.

The required sample size was estimated using the method [12] developed for comparing two independent proportions in a 2-sided test. Assumptions made for the sample size estimation are: the proportions of subjects with malaria in irrigated and pastoral study site were 20 and 10 %, respectively; the level of confidence that the difference between these proportions is not due to chance is 95 %; and the power of the study to find a difference in the prevalence is 80 %. This analysis was implemented in STATA using *sampsi* command, which indicated that at least 220 subjects were required in each site to meet the conditions set a priori.

### Malaria prevalence

Members of the households selected for the study were taken through the study design to show how they had been recruited for the survey. After this, they were requested to participate in the survey, with signed consents being obtained from the household head, the subject themselves and an independent observer who had to be a member of the community. Once the consent was obtained, blood samples were obtained from each subject (who was over 5 years old) within the selected households using a sterile vacutainer and butterfly needles from the median cubital veins. This was done by experienced phlebotomists and clinicians from the Ministry of Health. Venous blood was collected for purposes of screening a broader range of infections in another study where this study was embedded. A drop of the whole blood from each subject was then used to screen for Plasmodium infection using Carestart™ malaria HRP2 (pf) kit, a rapid diagnostic test (RDT). Participants with positive rapid tests were treated by the clinicians. Additional information for the each of the subjects were collected using an electronic form designed using open data kit (ODK) kit [13] and administered to every subject except for those under 18 years where the household head answered the questions on their behalf. The information included relationship to household head, age, sex, occupation, and self-reported malaria (“have you had malaria in the past 2 months?”).

### Community knowledge on risk factors and control practices for malaria

A structured questionnaire was administered to the heads of the same households that were screened for malaria immediately after blood sampling. The information gathered through the questionnaire included demographic data of the households, knowledge on malaria, control/prevention measures both for the disease and mosquito vectors and the treatment regimens for the clinical cases. Household characteristics such as house construction materials, activities of the occupants, source of drinking water and environmental factors in homesteads thought to influence human mosquito contact were also collected. Households and health facilities were also mapped. The data were collected using ODK collect software in a Samsung galaxy Tablet 7. Questionnaire used in the survey was pre-tested in a village outside the study site before the survey commenced.

### Ethical considerations

Ethical clearance was provided by the African Medical and Research Foundation-Ethics and Scientific Review Committee (AMREF-ESRC), number AMREF-ESRC P65/2013. All RDT confirmed cases of malaria were treated by a registered clinician.

### Data analysis

The QGIS version 2.0 was used to compute the vector distances from the household to the nearest health facility using coordinates for the households and health facilities. Data were cleaned and analysed in STATA version 12.0. Descriptive statistics were used to examine the data. The analysis of knowledge and practices data was done by generating proportions, frequencies and means. An immediate form of two-sample test of proportions was done using the command *prtesti* in STATA to determine whether the proportions in Bura and Ijara were statistically different.

A generalized linear model with a logit link was used to identify factors associated with malaria infection. Two response variables were used: (1) the RDT results, and (2) whether a subject reported that he or she had had malaria in the last two months before the survey. The second outcome was a self-reported observation captured by the questionnaire administered to the household head. After descriptive analysis, independent variables used comprised age, gender, household size and distance to the nearest health facility. Both univariate and multivariate models were fitted. The goodness-of-fit of the final model was tested using likelihood ratio test.

## Results

### Household demographics and characteristics

A total of 334 households participated in the knowledge and practices survey: 114 from Bura irrigation scheme and 220 from Ijara. Table 1 shows a comparison of the proportion of various attributes in Bura and Ijara including the household demographics and characteristics. 44 and 63.6 % of all the household respondents were males in Bura and Ijara, respectively and these proportions were not significantly different (p = 0.10). Generally, the occupation (except small business and casual labour) of household heads between the two sites differed significantly (p < 0.05): 79.8 % farmers and 27.2 % pastoralists in Bura irrigation scheme while there were no farmers in Ijara but 87.7 % were pastoralists. Almost all households (94.7 % in Bura and 91.8 % in Ijara; p = 0.33) kept at least one species of livestock within their homesteads. Some roofing and wall materials of the houses differed significantly (p < 0.05) between the two sites: use of iron sheets for roofing were mainly (63.2 %) in Bura while residents of Ijara mainly (81.4 %) used grass for roofing; the main wall material in Bura was mud and wood (53.5 %) while grass was the most common (67.3 %) wall material in Ijara; and use of canes/trunks as wall materials was more common in Ijara (15.9 %) compared to Bura (3.5 %). Other roofing materials (such as nylon papers and clothes) and wall materials (such as bricks, stone with mud and wood planks/shingles) were least common in both sites and were not significantly different (p > 0.05). Irrigation water canals were the major (70.2 %) source of water for domestic use in Bura while dams (57.7 %) and water pans (20.0 %) were mainly used in Ijara. These proportions were statistically different (p < 0.05) between the two sites.

**Table 1:** Distribution of household characteristics, and control and management strategies of malaria in Bura (N = 114) and Ijara (N = 220)

Variable	Levels	Bura irrigation		Ijara pastoral		Proportion test
		n	%	n	%	p value*
Respondent’s sex	Male	62	54.4	140	63.6	0.10
	Female	52	45.6	80	36.4	0.10
Occupation of household head	Farmer	91	79.8	0	0.0	0.00
	Pastoralist	31	27.2	193	87.7	0.00
	Small business	4	3.5	9	4.1	0.80
	Casual labour	2	1.8	3	1.4	0.78
Animal kept within compound	Yes	108	94.7	202	91.8	0.33
	No	6	5.3	18	8.2	0.33
Main floor material	Earth	108	94.7	198	90.0	0.14
	Cement	6	5.3	13	5.91	0.81
Main roof material	Iron sheets	72	63.2	17	7.73	0.00
	Grass	30	26.3	179	81.4	0.00
	Others such as nylon papers, clothes, no roof	12	10.5	24	10.9	0.91
Main wall material	Mud and wood	61	53.5	4	1.8	0.00
	Grass	22	19.3	148	67.3	0.00
	Stone with cement	9	7.9	7	3.2	0.06
	Cane/trunks	4	3.5	35	15.9	0.00
	Others like bricks, stone with mud and wood planks/shingles	8	7.0	26	11.8	0.17
Main water source	Water canals	80	70.2	0	0.0	0.00
	Dams	27	23.7	127	57.7	0.00
	Protected well	3	2.6	15	6.8	0.11
	Water tankers	3	2.6	0	0.0	0.02
	Water pans	0	0.0	44	20.0	0.00
Heard of malaria	Yes	114	100.0	217	98.6	0.21
	No	0	0.0	3	1.4	0.21
Cause of malaria	Mosquito bites	109	95.6	215	97.7	0.28
	Dirty home surroundings	11	9.7	29	13.2	0.35
	Stagnant water	7	6.1	79	35.9	0.00
	Taking dirty water	11	9.7	28	12.7	0.42
	Others such as dam water, long rains, eating raw food and cold/wet weather	14	12.3	51	23.2	0.02
Malaria transmission	Infectious mosquito bites	35	30.7	9	4.1	0.00
	Contact with sick person	4	3.5	0	0.0	0.01
	Others like taking contaminated water	2	1.8	3	1.4	0.78
	Not transmitted	73	64.0	208	94.6	0.00
Malaria symptoms	Fever	100	87.7	213	96.8	0.00
	Headache	86	75.4	139	63.2	0.02
	Muscle and joint pain	57	50.0	84	38.2	0.04
	Chills/shivering	53	46.5	163	74.1	0.00
	Vomiting	51	44.7	108	49.1	0.45
	Body weakness/fatigue	46	40.4	87	39.6	0.89
	Loss of appetite	38	33.3	109	49.6	0.00
Access to a health facility	Yes	110	96.5	200	90.9	0.06
	No	4	3.5	20	9.1	0.06
Distance to the nearest health facility	≤5 km	57	50.0	120	54.5	0.43
	>5 km	57	50.0	100	45.5	0.43
Action taken within 1 day of malaria symptoms	Taken to hospital	22	29.3	30	34.1	0.38
	AL self-medication at home	15	20.0	3	3.4	0.00
	Paracetamol self-medication	14	18.7	11	12.5	0.13
	Nothing	13	17.3	19	21.6	0.36
Drugs taken	AL	53	46.5	28	12.7	0.00
	Paracetamol	28	24.6	19	8.6	0.00
	Quinine	17	14.9	11	5.0	0.00
	Fansidar	8	7.0	36	16.4	0.02
Source of health care	Government health facility	88	77.2	196	89.1	0.00
	Private health facility	22	19.3	3	1.4	0.00
	No health care	4	3.5	20	9.1	0.06
Main source of drugs taken at home	Pharmacy/chemist	18	69.2	12	46.2	0.00
	Ordinary shops	5	19.2	11	42.3	0.00
Malaria prevention/control	Use of mosquito nets	108	94.7	158	71.8	0.00
	Clearing of bushes and vegetation around houses and canals	18	15.8	51	23.2	0.11
	Draining/leveling of breeding sites around house	16	14.0	43	19.6	0.21
	Indoor residual spraying	6	5.3	4	1.8	0.08
	Taking drugs	5	4.4	139	63.2	0.00
	Lighting fires and mosquito coils	4	3.5	18	8.2	0.10
All members used mosquito net last night	Yes	99	86.8	39	17.7	0.00
	No	15	13.2	181	82.3	0.00
Sources of mosquito nets	Government health centre	75	65.8	31	14.1	0.00
	Government campaigns	27	23.7	8	3.6	0.00
	Non-governmental organizations	15	15.8	7	3.2	0.00
	Retail shops	9	7.9	67	30.5	0.00
	Chemists	3	3.6	0	0.0	0.00
	Private clinics	2	2.4	5	2.4	0.99
Indoor residual sprays in the last 12 months	Yes	33	29.0	1	0.5	0.00
	No	81	71.0	219	99.5	0.00

*AL* artemether–lumefantrine

* Two sample test of proportions

### Community knowledge of malaria disease

Results for the community knowledge of malaria in Bura and Ijara are shown in Table 1. Overall, similar results of respondents having prior information on malaria in Bura irrigation scheme and pastoral Ijara were reported (100 % in Bura, 98.6 % in Ijara; p = 0.21). Similarly, there was no significant difference (p < 0.05) in most responses for the causes of malaria: mosquito bites were reported by 95.6 % of respondents in Bura and 97.7 % in Ijara; while other responses included dirty water surroundings (9.7 % in Bura, 13.2 % in Ijara) and taking dirty water (9.7 % in Bura, 12.7 % in Ijara). More respondents in Ijara than in Bura reported stagnant water (35.9 vs 6.1 %, p < 0.01) and other responses for the causes of malaria such as dam water, long rains, eating raw food, and cold weather (23.2 vs 12.3 %; p = 0.02). A significant difference (p < 0.05) in the knowledge of malaria transmission by respondents in Bura and Ijara were reported: 30.7 % of the respondents in Bura reported infectious mosquito bites as the mode of transmission while only 4.1 % gave a similar response in Ijara; 3.5 % of the respondents in Bura identified contact with sick person as a mode of transmission while none in Ijara gave that response; and notably, 64.0 and 94.6 % of the respondents in Bura and Ijara respectively, reported that malaria isn’t transmitted. A significant difference on the symptoms of malaria was reported between Bura and Ijara: fever (87.7 vs 96.8 %; p < 0.01); headache (75.4 vs 63.2; p = 0.02); muscle and joint pain (50 vs 38.2 %; p = 0.04); chills/shivering (46.5 vs 74.1 %; p < 0.01); vomiting (44.7 vs 49.1 %; p = 0.45); and loss of appetite (33.3 vs 49.6 %; p < 0.01). Body weakness/fatigue was equally reported by respondents in both sites (40.4 % in Bura vs 39.6 % in Ijara; p = 0.89).

### Case management of malaria

Table 1 includes results for the case management of malaria in Bura and Ijara. Having access to a health facility was similarly reported in both sites (Bura 96.5 %, Ijara 90.9 %; p = 0.06). The average vector distance a malaria victim travelled to the nearest health facility in Bura irrigation scheme was 7.2 km while those in Ijara traveled an average distance of 6.9 km. There was no statistical difference (p = 0.43) between those who travelled ≤5 km in Bura (50 %) and in Ijara (54.6 %) to the nearest health facility. In both sites, most respondent reported mainly going to the hospital within one day of malaria symptoms (Bura 29.3 %, Ijara 34.1 %; p = 0.38). Self-medication of artemether–lumefantrine (AL) within one day of malaria symptoms was highly (p < 0.01) reported in Bura (20 %) compared to Ijara (3.4 %). Paracetamol self-medication within the first day of malaria symptoms was equally practiced in both sites (Bura 18.7 %, Ijara 12.5 %; p = 0.13). Similarly, some respondents reported doing nothing following malaria symptoms within the first day in both sites (Bura 17.3 %, Ijara 21.6 %; p = 0.36). Government health facility was reported as the main source of health care in both sites and mainly in Ijara (Bura 77.2 %, Ijara 89.1 %; p < 0.01). Private health facilities were mainly used in Bura (19.3 %) compared to Ijara (1.4 %); p < 0.01. A significant difference in usage of reported common malaria drugs was observed: AL (Bura 46.5 %, Ijara 12.7 %; p < 0.01); Paracetamol (Bura 24.6 %, Ijara 8.6 %; p < 0.01); Quinine (Bura 14.9 %, Ijara 5.0 %; p < 0.01); and Fansidar (Bura 7.0 %, Ijara 16.4 %; p = 0.02). Differences in sources of drugs for self-medication at home between the two sites were also reported: pharmacy/chemist (Bura 69.2 %, Ijara 46.2 %; p ≤ 0.01); ordinary shops (Bura 19.2 %, Ijara 42.3 %; p < 0.01) of the respondents in Bura reported obtaining the drugs from the local pharmacy/chemist while 46.2 %.

### Control and prevention of malaria

Respondents reported that they used a variety of measures to control and prevent malaria in their homes (Table 1). Use of mosquito nets was mainly reported in both sites but more significantly in Bura irrigation scheme (Bura 94.7 %, Ijara 71.8 %; p < 0.01). Additionally, a significantly (p < 0.01) higher proportion of respondents in Ijara (63.2 %) reported taking drugs to control/prevent malaria compared to those in Bura (4.4 %). Other malaria control/prevention methods that were used in equal measures included: clearing of bushes and vegetation around houses and canals (Bura 15.8 %, Ijara 23.2 %; p = 0.11); draining/leveling of breeding sites around houses (Bura 14.0 %, Ijara 19.6 %; p = 0.21); indoor residual spraying (Bura 5.3 %, Ijara 1.8 %; p = 0.08); and lighting fires and mosquito coils (Bura 3.5 %, Ijara 8.2 %; p = 0.10). Generally, residents of Bura mainly obtained mosquito nets from government health centres (65.8 %) and government campaigns (23.7 %) while those from Ijara obtained the nets mainly from retail shops (30.5 %). Other sources of mosquito nets included: non-governmental organizations (Bura 15.8 %, Ijara 3.2 %; p < 0.01); chemists (Bura 3.6 %, Ijara 0.0 %, p < 0.01); and private clinics (Bura 2.4 %, Ijara 2.4 %; p = 0.99). In Bura irrigation scheme, the average number of nets per household was three (average household size of 6) while in Ijara an average of one net per household (average household size of 7) was reported. A significantly (p < 0.01) higher usage of mosquito nets the previous night by all members of household was reported in Bura (86.8 %) compared to Ijara (17.7 %). There was no strong evidence of association between number of nets in a household and vector distance (>5 km compared to ≤5 km; estimate = −0.27; p = 0.17) to the nearest health facility. A significantly (p < 0.01) higher usage of indoor residual sprays was reported in Bura (29.0 %) compared to Ijara (0.5 %).

### Malaria prevalence

Using Carestart™ malaria HRP2 (pf) kit, eight out of 171 people tested positive (prevalence of 4.68 %; 95 % confidence interval of 1.48 and 7.88 %) in Bura irrigation scheme while only one out of 322 people tested positive (prevalence of 0.31 %; 95 % confidence interval of −0.30 and 0.92 % in Ijara. However, 14.62 % (95 % confidence interval: 9.27–19.97 %) of individuals in Bura reported having had malaria within a period of 2 months before implementation of this study compared to 23.91 % (95 % confidence interval: 19.23–28.60 %) with a similar response in Ijara.

Table 2 shows the distribution of RDT positive and self-reported malaria by age group and site. Individuals who were over 18 years or older had higher prevalence of Plasmodium infection (Bura 3.6 %, Ijara 0.4 %) as well as self-reported malaria (Bura 21.6 %, Ijara 31.9 %) compared to younger individuals.

**Table 2:** Distribution of RDT result and self-reported malaria by age groups and site

Site	Age group (years)	No. screened	RDT positive	Self-reported malaria
		N	n (%)	n (%)
Bura irrigation scheme	<12	33	2 (6.1)	0 (0.0)
	12–17	27	2 (7.4)	1 (3.7)
	≥18	111	4 (3.6)	24 (21.6)
Pastoral Ijara	<12	71	0 (0.0)	2 (2.8)
	12–17	25	0 (0.0)	3 (12.0)
	≥18	226	1 (0.4)	72 (31.9)

### Risk factors associated with malaria based on RDT results

Table 3 shows the results of univariate and multivariable logistic regression analysis of the association between RDT result and the potential explanatory variables. A significantly lower prevalence of Plasmodium infection was reported amongst people in Ijara (OR = 0.06; p = 0.01) compared to those in Bura irrigation scheme. At 90 % level of confidence, the multiple logistic regression showed that people who lived more than 5 km to the nearest health facility (OR = 4.05; p = 0.09) had higher prevalence of Plasmodium infection compared to people who lived within 5 km vector distance from the nearest health facility. Age, gender and size of the household were not significantly associated with Plasmodium infection.

**Table 3:** Logistic regression with RDT result as the response variable

Variable	Univariate		Multivariate	
	OR (95 % CI)	p value	OR (95 % CI)	p value
*Site*				
Irrigated (reference)				
Pastoral	0.06 (0.01–0.51)	0.01	0.05 (0.01–0.40)	0.01
Age of individual	0.66 (0.28–1.57)	0.35	0.61 (0.25–1.51)	0.28
*Sex of individual*				
Female (reference)				
Male	1.38 (0.36–5.19)	0.64	1.08 (0.27–4.35)	0.92
Size of household	0.87 (0.69–1.08)		0.92 (0.70–1.22)	0.58
*Distance to the nearest health facility*				
<5 km (reference)				
>5 km	1.63 (0.34–7.95)	0.54	4.05 (0.78–21.05)	0.09

### Risk factors associated with self-reported malaria

The result of the univariate and multivariable logistic regression with the outcome being whether an individual had suffered from malaria within the last 2 months before the implementation of this study are shown in Table 4. People from the pastoral Ijara were twice more likely (OR = 2.32; p = 0.01) to report having had malaria within the last 2 months before this study compared to those from the irrigated area (Bura). Furthermore, for a unit increase in age of the respondent, the odds of having a person who had perceived malaria infection within the last 2 months of this study increased by about five times (OR = 4.75; p < 0.01).

**Table 4:** Logistic regression for self-reported malaria

Variable	Univariate		Multivariate	
	OR (95 % CI)	p value	OR (95 % CI)	p value
*Site*				
Irrigated (reference)				
Pastoral	1.84 (1.12–3.01)	0.02	2.32 (1.29–4.20)	0.01
Age of individual	4.50 (2.95–6.87)	0.00	4.75 (3.07–7.36)	0.00
*Sex of individual*				
Female (reference)				
Male	1.13 (0.72–1.77)	0.59	1.24 (0.75–2.04)	0.39
Size of household	1.03 (0.97–1.09)	0.34	0.99 (0.94–1.06)	0.98
*Distance to the nearest health facility*				
<5 km (reference)				
>5 km	1.14 (0.71–1.84)	0.59	0.87 (0.50–1.52)	0.63

## Discussion

Malaria is a major public health problem worldwide and many countries continue to experience heavy mortalities and economic losses due to ill health. The World Health Organization (WHO) indicates that a total of US Dollars (US$) 5.1 billion is required to meet the global targets for the control and elimination of the disease [14]. Such intervention programs would benefit from studies that characterize potential risk in order to prioritize areas or practices for action. In this study, the prevalence and associated risk factors of the disease as well as knowledge on control and management practices that were being used by the local communities in Bura irrigation scheme and the pastoral Ijara in Kenya were identified as part of a larger study that determined linkages between land use change and mosquito-borne infections.

Multivariable analyses based on the RDT result showed that people living in Bura irrigation scheme were more likely to be infected compared to those living in the pastoral area of Ijara. Irrigation schemes are known to create suitable breeding sites for mosquito that transmit the malaria parasites [3–6]. Households located less or equal to 5 km to the nearest health facility had lower prevalence of Plasmodium infection compared to those located more than 5 km away. Similar finding was made by Larson et al. [15] in Malawi and they attributed the lower infection rate in households close to health facilities to availability of more mosquito nets compared to those further away. However, we found a weak association between mosquito nets and vector distance to the nearest health facility. Possible explanations for this finding would include the likelihood that environment, vegetation, housing construction and condition, water drainage as well as health information may vary between areas close to health centres versus those further away.

Analysis that used perceived malaria exposure at individual level as the outcome indicated that people living in non-irrigated area of Ijara were twice more likely to report having suffered from malaria within the last 2 months before the implementation of this study compared to those living in Bura irrigation scheme. This might indicate the possibility of misdiagnosis of other febrile illness leading to exaggerated treatment for malaria. Significantly high prevalence of Q fever and West Nile virus are being reported by an ongoing project (dynamic drivers of diseases in Africa: ecosystems, livestock/wildlife, health and wellbeing conducted by International Livestock Research Institute) yet the community does not know about these other febrile diseases. A unit increase in age increased the odds of having a person who had perceived malaria infection within the last 2 months of this study by about five times indicating possibilities of misdiagnoses of other febrile diseases especially to the older age groups.

Contrary to other studies [16, 17], age, sex and size of household were not significantly associated with Plasmodium infection. Other potential factors such as housing conditions, livestock ownership, number of nets and occupation were not included in the model since they are site-specific and thus a representation of the two study areas. The housing conditions in most of the households visited in Bura irrigation for instance consisted of muddy walled houses roofed with old and rusting iron sheets while those houses in Ijara were mainly constructed using grass. Other previous studies have shown that residential house status or structure influence malaria transmission as poorly constructed houses expose individuals to mosquitoes by providing suitable resting places or shelter and therefore attacking the occupants [18]. Yé et al. [19] observed that, children living in houses with mud roofs had significantly higher risk of getting malaria infection compared to those living in iron-sheet roofed houses. A possible explanation for this scenario is that iron sheet roof is not a suitable resting place for blood-engorged mosquitoes as opposed to cracks in the mud roof.

The community members in both Bura irrigation scheme and Ijara demonstrated good knowledge on malaria—its causes, symptoms, treatment and prevention by using bed nets. Insecticide treated nets (ITNs) are the most practical method of mosquito control that are used to protect at-risk individuals from mosquito bites and hence malaria infections [20]. Through interviews, the community members in Bura irrigation scheme revealed a very high ownership and usage of mosquito nets regardless of whether it is treated or not. The use of untreated mosquito nets has been found to have some protective measures against mosquito bites while ITNs increases the mortality of vectors in addition to reducing vector-host contact, and therefore reducing the transmission of malaria [21].

In this study, the major sources of the bed nets were the government health facility and government campaigns both of which could have resulted in the observed overwhelming possession and use of bed nets by the community. In 2007, the Mentor Programme with the support from Department for International Development (DFID) distributed 17,700 long-lasting insecticide-treated nets (LLIN) to pre-empt the malaria epidemic effects of flooding in Tana River and Garissa Districts [22]. Free distribution of ITNs in Kenya also takes place through antenatal and child welfare clinics to pregnant women and children under 1 year of age and through comprehensive care clinics for people living with human immunodeficiency virus (HIV). In 2009, a global strategy of ensuring universal coverage with ITNs (one net for every two people) for all persons at risk of malaria was adopted by the government of Kenya [23]. This target seems to have been met in Bura irrigation scheme. According to the 2015 Kenya malaria indicator survey, an increased uptake in ownership and use of mosquito nets as well as improved utilisation of the recommended malaria drugs were reported countrywide.

Only a few households practiced environmental management such as bush clearing around homesteads and draining of stagnant water as methods of controlling malaria. Environmental management has been shown [24] to be a cost-effective method of reducing mosquito abundance which, if coupled with other vector and malaria parasite control methods, would substantially reduce malaria. These constitute an integrated malaria management (IMM) package and have been shown to be very successful in many tropical environments [25]. Okech et al. [26] observed that, implementation of IMM in a community in Mwea irrigation scheme in Kenya could have led to a drastic fall in malaria incidence. Thus, there is a need for the residents of Bura irrigation scheme and Ijara to be educated on other methods of malaria control if the prevalence of the disease is to be further reduced.

Government health centers are a backbone in case management of malaria. In the study sites, the major government facilities were Bura and Ijara Health Centres and the drug of choice in the treatment of malaria was artemether–lumefantrine (AL). This is the recommended drug for malaria in Kenya by the Ministry of Health since 2006 after increasing resistance levels to SP. The AL is freely available in government and faith-based health facilities and since its introduction the trends of malaria infection cases in Kenya have been decreasing. Most people in Bura irrigation scheme were aware about the drug and its use. It is also worth noting that the use of SP was relatively high in Ijara an indication of self-medication at home by the community.

## Conclusions

The communities in Bura irrigation schemes and Ijara demonstrated relatively good knowledge on causes, transmission and control/prevention of malaria. However, awareness is required on proper diagnosis and management of the disease especially in Ijara. People residing in Bura irrigation scheme were more likely to be infected compared to those living those living in the pastoral area of Ijara. However, the community in Ijara was more likely to be treated for perceived malaria infection which could otherwise be confused for other febrile illnesses. To ensure proper diagnosis, screening of these other febrile diseases is therefore required in this community. Households located nearer to the health facilities had a lower prevalence of malaria than households located far away indicating the importance of health services in malaria control.

## Abbreviations

AL: artemether–lumefantrine
AMREF-ESRC: African Medical and Research Foundation-Ethics and Scientific Review Committee
DFID: Department of International Development
DOMC: Division of Malaria Control
HIV: human immunodeficiency virus
IMM: integrated malaria management
IRS: indoor residual spraying
ITNs: insecticide-treated nets
LLIN: long-lasting insecticidal net
ODK: open data kit
RDT: rapid diagnostic test
SP: sulfadoxine–pyrimethamine
US$: US Dollars
WHO: World Health Organization
